# Inspiration for the Future: The Role of Inspiratory Muscle Training in Cystic Fibrosis

**DOI:** 10.1186/s40798-019-0210-3

**Published:** 2019-08-08

**Authors:** Ren-Jay Shei, Robert L. Dekerlegand, Kelly A. Mackintosh, John D. Lowman, Melitta A. McNarry

**Affiliations:** 1 Division of Pulmonary, Allergy, and Critical Care Medicine, Department of Medicine, University of Alabama at Birmingham, 1918 University Boulevard, Birmingham, AL 35294-0006 USA; 2 Gregory Fleming James Cystic Fibrosis Research Center, University of Alabama at Birmingham, Birmingham, AL USA; 3 Department of Physical Therapy, College of Rehabilitation Sciences, Jefferson (Philadelphia University and Thomas Jefferson University), Philadelphia, PA USA; 4 Applied Sports Science Technology and Medicine Research Centre (A-STEM), College of Engineering, Swansea University, Swansea, UK; 5 Department of Physical Therapy, School of Health Professions, University of Alabama at Birmingham, Birmingham, AL USA

## Abstract

Cystic fibrosis (CF) is an inherited, multi-system, life-limiting disease characterized by a progressive decline in lung function, which accounts for the majority of CF-related morbidity and mortality. Inspiratory muscle training (IMT) has been proposed as a rehabilitative strategy to treat respiratory impairments associated with CF. However, despite evidence of therapeutic benefits in healthy and other clinical populations, the routine application of IMT in CF can neither be supported nor refuted due to the paucity of methodologically rigorous research. Specifically, the interpretation of available studies regarding the efficacy of IMT in CF is hampered by methodological threats to internal and external validity. As such, it is important to highlight the inherent risk of bias that differences in patient characteristics, IMT protocols, and outcome measurements present when synthesizing this literature prior to making final clinical judgments. Future studies are required to identify the characteristics of individuals who may respond to IMT and determine whether the controlled application of IMT can elicit meaningful improvements in physiological and patient-centered clinical outcomes. Given the equivocal evidence regarding its efficacy, IMT should be utilized on a case-by-case basis with sound clinical reasoning, rather than simply dismissed, until a rigorous evidence-based consensus has been reached.

## Key Points

- Preliminary evidence indicates that inspiratory muscle training could enhance respiratory muscle function in CF, although whether this translates to meaningful physiological and patient-centered improvements is unclear.
- The poor quality of evidence limits the conclusions that can be drawn and highlights the need for future studies that utilize appropriate measures and training protocols and characterize the patient population to enable conclusions to be drawn regarding the potential therapeutic benefits of IMT in CF.
- Until such studies have been conducted, conclusions on the value of IMT as a treatment tool in CF cannot be drawn and as such, IMT should be considered on a case-by-case basis.

## Introduction

Cystic fibrosis (CF) is an autosomal recessive genetic disorder caused by mutations in the cystic fibrosis transmembrane conductance regulator (*CFTR*) gene that results in multi-organ pathologies involving the respiratory, digestive, and reproductive systems [1–3]. Specific to the respiratory system, aberrant or absent CFTR channels produce a dehydrated, hyper-viscous, and acidic mucosal layer that promotes an airway environment prone to chronic infection and inflammation that leads to progressive lung injury. Airway obstruction becomes evident in pulmonary function tests as does a progressive lung hyperinflation associated with a reduction in forced expiratory volume in 1 s (FEV_1_) [4, 5]. The resultant increased work of breathing (WOB) and decreased gas exchange contribute to impaired exercise capacity and ultimately respiratory failure, which is the leading cause of CF-related mortality [1, 2].

Alterations in pulmonary mechanics associated with progressive CF pathophysiology include the adoption of a rapid, shallow breathing pattern [6], which may delay respiratory muscle fatigue [7], but also contribute to increased WOB and impaired gas exchange [6, 8]. Multiple CF-related comorbidities, including thoracic kyphosis and postural abnormalities, can create a restrictive lung dysfunction that elevates the demand imposed on the respiratory pump [9, 10]. Thus, individuals with CF commonly exhibit an imbalance between the ventilatory load and the capacity of the respiratory muscles, which is exemplified by the adoption of inefficient breathing patterns [6] and exercise intolerance [11].

Although alterations in pulmonary mechanics and WOB in individuals with CF are clear, it is equivocal whether individuals with CF exhibit respiratory (inspiratory and/or expiratory) muscle dysfunction or preserved respiratory muscle function [12, 13]. It is evident, however, that respiratory muscle function is influenced by a variety of factors including hyperinflation, nutritional status [14], systemic corticosteroid use, *Pseudomonas aeruginosa* colonization, inactivity, and chronic inflammation [15]. Importantly, respiratory muscle performance in CF is a key determinant of aerobic fitness [15], which is closely associated with survival [16] and quality of life (QoL) [17–19]. Thus, addressing whether inspiratory and expiratory muscle function is preserved or aberrant in CF will be important to identify novel methods to optimize respiratory pump function in CF, including inspiratory muscle training (IMT).

Strategies to enhance respiratory muscle function in the presence of a load/capacity imbalance and to counteract the disease-related decline in pulmonary function have been suggested, with one potential strategy being IMT [20, 21]. While specific loading protocols vary, IMT typically utilizes either a pressure or volume load on the inspiratory muscles to provide a stimulus to elicit a training response [22], similar to that observed in response to training peripheral musculature. Indeed, IMT has been shown to be an effective ergogenic aid to enhance exercise performance in healthy adults [23–26] and has been investigated as a therapeutic intervention to improve clinical and functional outcomes in a variety of health conditions including asthma, chronic obstructive pulmonary disease (COPD), heart failure, and stroke [27–33]. However, the applicability of IMT as a therapeutic strategy in those with CF remains equivocal [20]. Nonetheless, whilst recent reviews have provided a comprehensive summary of the current evidence base, little attention has been given to the methodological limitations associated with this evidence, which largely confounds its interpretation and should temper any conclusions. Therefore, the purpose of the present opinion paper is to draw attention to past and ongoing challenges with IMT studies and to provide recommendations on how future studies may seek to address such shortcomings to provide a better understanding of how IMT may, or may not, fit into the armamentarium of tools to manage CF.

## Current State of IMT in CF

A summary of peer-reviewed publications on IMT in individuals with CF is given in Table 1. Despite the rehabilitative and therapeutic potential of IMT, its clinical efficacy and long-term application in CF remains inconclusive as no study to date has provided strong evidence of significant improvements in clinical outcomes, regardless of improvements in inspiratory muscle performance. It should be noted, however, that IMT has been associated with improved exercise tolerance [34] and improved health-related QoL within the mastery and emotion domains [34, 35]. The apparent limited utility of IMT to translate to clinically meaningful outcomes must be interpreted in the context of multiple methodological limitations and the obvious paucity of available studies. Indeed, the most recent Cochrane review only identified nine reports eligible for inclusion [20]. Whilst the overall conclusion of this review appears to be appropriate based on the available evidence, several parameters that may underpin the equivocal findings were not adequately discussed, such as the methods utilized to quantify respiratory muscle function [36]. Furthermore, little distinction was made between children and adults within this recent review, despite significant physiologic and psychosocial age-related differences that may impact the plasticity and response to IMT. This observation is of particular concern given the inherent interaction between age and disease progression in CF, which is highly likely to influence the efficacy of any intervention. The lack of available data limits child-adult comparisons, and although Hilton et al. [20] proposed an arbitrary 16-year-old cut-point to distinguish adults, this threshold may be inappropriate given the delayed biological maturation that may occur with CF [37, 38].

**Table 1:** Summary matrix of original published studies investigating inspiratory muscle training in individuals with cystic fibrosis

Study	Patient demographics*	Protocol	Comparison	Primary outcomes
Asher, 1982	Age: 16.0 ± 4.6 %BMI: 82.6 ± 9.9 %FEV_1_: 35.0 ± 12.3 MIP: 74 ± 18	Mode: Flow-based Intensity: *R*_max_ Frequency: BID Duration: 15 min/day; 4 weeks	Subjects served as their own controls with a 4-week control period followed by a 4-week intervention period.	Increase in IMS (9.5%; *p* < 0.025) and IME; no effect on exercise performance.
Sawyer, 1993	Age: 11.5 ± 2.5 BMI**: 18.4 NIHS: 87.7 MIP: 107 ± 29	Mode: Threshold Intensity: 50–60% MIP Frequency: 7 days/week Duration: 30 min/day; 10 weeks	Compared to a sham group who performed trained at ≤ 10% MIP.	Increase in MIP (13%; *p* < 0.01), VC (17%), TLC (13%; *p* < 0.01), and maximal exercise capacity (9.8%; *p* < 0.03) with observed increase in sputum production.
De Jong, 2001	Age: 17 ± 5.2 BMI**: 17.9 %FEV_1_: 70 ± 25 %MIP: 105 ± 23	Mode: Threshold Intensity: 40% MIP Frequency: 5 days/week Duration: 20 min; 6 weeks	Compared to a sham group who performed trained at 10% MIP.	Increase in IME (35%; *p* = 0.003) with no significant effect on exercise, dyspnea, or fatigue.
Enright, 2004	Age: 24.8 ± 5.5 BMI**: 22.3 %FEV_1_: 64.2 ± 29.7 MIP: 134 ± 26	Mode: Computer interface Intensity: 80% SMIP Frequency: 3 days/week Duration: 6 sets, 6 reps; 8 weeks	Compared to a sham group at 20% SMIP and a control group.	Increased SMIP and MIP with 80% and 20% training groups with no between group differences. Increased diaphragmatic thickness (20%), VC (24%), TLC (12%), and PWC (51%); decreased anxiety and depression in the 80% group only.
Santana-Sosa, 2014	Age: 11 ± 1 BMI: 16.6 ± 0.7 FEV_1_: 1.65 ± 0.19 MIP: 68.3 ± 6.3	Mode: Threshold combined with exercise program. Intensity: 40–50% MIP Frequency: BID Duration: ~ 5 min of 30 inspirations; 8 weeks	Compared to a sham group who was trained at 10% MIP.	Increased MIP (58%), VO_2_peak (22%), and muscular strength in the intervention group.
Bieli, 2017	Sequence IC*** Age: 15.4 (12.0:16.6) BMI**: 17.8 zFEV_1_: − 0.9 (− 2.8:0.5) MIP: Not reported Sequence CI*** Age: 13.2 (10.9:17.8) BMI**: 19.7 zFEV_1_: − 2.1 (− 3.4 : - 0.5) MIP: Not reported	Mode: Eucapnic hyperventilation Intensity: Not reported Frequency: BID, 5 days/week Duration: 10 min; 8 weeks	Randomized crossover comparison.	Increased RME (105%) but not exercise endurance, lung function, or quality of life.

*BID* two times per day, *BMI* body mass index, *%BMI* body mass index percentile, *%FEV*_*1*_ percent of predicted forced expiratory volume in 1 s, *FEV*_*1*_ forced expiratory volume in 1 s expressed as liters per second, *zFEV*_*1*_ forced expiratory volume in 1 s expressed a *z*-score, *%RV* percent of predicted residual volume, *R*_*max*_ greatest resistance sustainable for 10 min, *IMS* inspiratory muscle strength, *IME* inspiratory muscle endurance, *%MIP* percent of predicted maximal inspiratory pressure, *MIP* maximal inspiratory pressure in cmH_2_O, *NIHS* National Institutes of Health Score for disease severity, *NR* not reported, *QD* daily, *PWC* physical work capacity, *SMIP* sustained maximal inspiratory pressure, *TLC* total lung capacity, *VC* vital capacity, *VO*_*2*_ *peak* peak rate of oxygen consumption

*Patient demographics given for baseline characteristics of the intervention group

**Calculated from height and weight provided in the article

***Values presented as median (interquartile range); *CI* control—intervention sequence, *IC* intervention—control sequence

The conflicting and uncertain results on the efficacy of IMT in CF may also be partially explained by variations in training protocols between studies [21]. Indeed, the contention that flow-based and threshold modes of IMT provide a similar training stimulus to the muscles, as cited in the recent Cochrane review [20], is controversial. Specifically, threshold devices do not provide a constant challenge throughout the inspiratory effort, while flow-based training is dependent on flow rates and breathing pattern [39]. In accord with skeletal muscle adaptations to training, it is vital to identify the optimal training protocol prior to conclusions being drawn regarding the efficacy of any training stimulus. At least in healthy humans, the efficacy of IMT as an ergogenic aid appears to be optimal when the IMT loading protocol is closely matched to the ventilatory demands of the criterion task (i.e., exercise modality) [24, 25], which has not been identified in CF. Indeed, the optimization of IMT is likely to be a highly individualized process requiring the IMT protocol to be matched with specifically identified respiratory muscle impairments. In this manner, an appropriate IMT protocol targeting strength and/or endurance deficits based on the individual’s needs may elicit optimal physiological benefits. However, despite their potential impact on intervention efficacy, these considerations have not been rigorously controlled for in prior studies on IMT in CF.

Further to resolving these methodological issues, the potential mechanisms underpinning the ability of IMT to elicit meaningful physiological and psychosocial benefits should be considered. It is possible that the apparent variability in the effect of IMT reflects inter-participant variations in disease manifestation and management that are of significant clinical and functional relevance, especially as the drive towards personalized/precision medicine continues [40, 41].

### Inspiratory Muscle Training: Potential Mechanistic Basis for Adaptations

The specific mechanisms by which IMT can transfer to meaningful outcomes in individuals with CF are unclear and likely multifactorial. However, several putative mechanisms have emerged, including (i) hypertrophy of the diaphragm and external intercostal muscles, (ii) an increase in the proportion of type I fibers in the external intercostal muscles, (iii) improvement in respiratory muscle economy and efficiency, (iv) reduction in the work of breathing, (v) enhanced respiratory muscle strength and endurance, (vi) attenuation of the respiratory muscle metaboreflex, (vii) reduction in cytokine release, (viii) reorganization of respiratory muscle motor recruitment pattern, (ix) decreased inspiratory muscle motor drive with preserved inspiratory pressure generation, and (x) decreased rating of perceived breathlessness and/or rating of perceived exertion [24, 42]. These mechanisms are likely interrelated and not mutually exclusive. Their interaction, and ultimately their impact on exercise performance, is not well understood. Whilst these potential mechanisms have been identified in healthy populations and their applicability to clinical populations, such as CF, remains to be elucidated, there are no grounds to postulate that the mechanisms may differ, although the emphasis on each factor may be disease-specific. For example, a reduction in cytokine release may be especially beneficial in a disease characterized by systemic inflammation, further highlighting the potential utility of IMT if shown to be effective.

## Expert Opinion on IMT in CF

Although IMT has the potential to be a useful rehabilitative and therapeutic tool for the management of CF lung disease, several crucial weaknesses in the current body of literature must be addressed in order to clarify its degree of utility. The grading of the evidence for IMT in CF as “very low quality” in the most recent Cochrane systematic review [20] highlights the challenges of conducting IMT studies in those with CF. The heterogeneity *between* and *within* studies, including wide variations in methodologies and outcome measures, the incomplete reporting of important parameters such as clinical status, and in the methods of randomization, allocation, and blinding [20], preclude firm conclusions to support or refute the use of IMT in this population. Indeed, methodologically rigorous studies that accurately and completely report these factors are required to come to a valid, evidence-based, consensus regarding the therapeutic efficacy of IMT in individuals with CF. The primary challenges within studies of IMT in those with CF that need to be addressed include (i) adequate consideration and controlling of the characteristics of the *patient population*, (ii) carefully targeting the *IMT protocol* based on individual needs, and (iii) selecting *outcome measures* that best capture both physiological adaptations and patient-centered clinical outcomes.

**Fig. 1:**
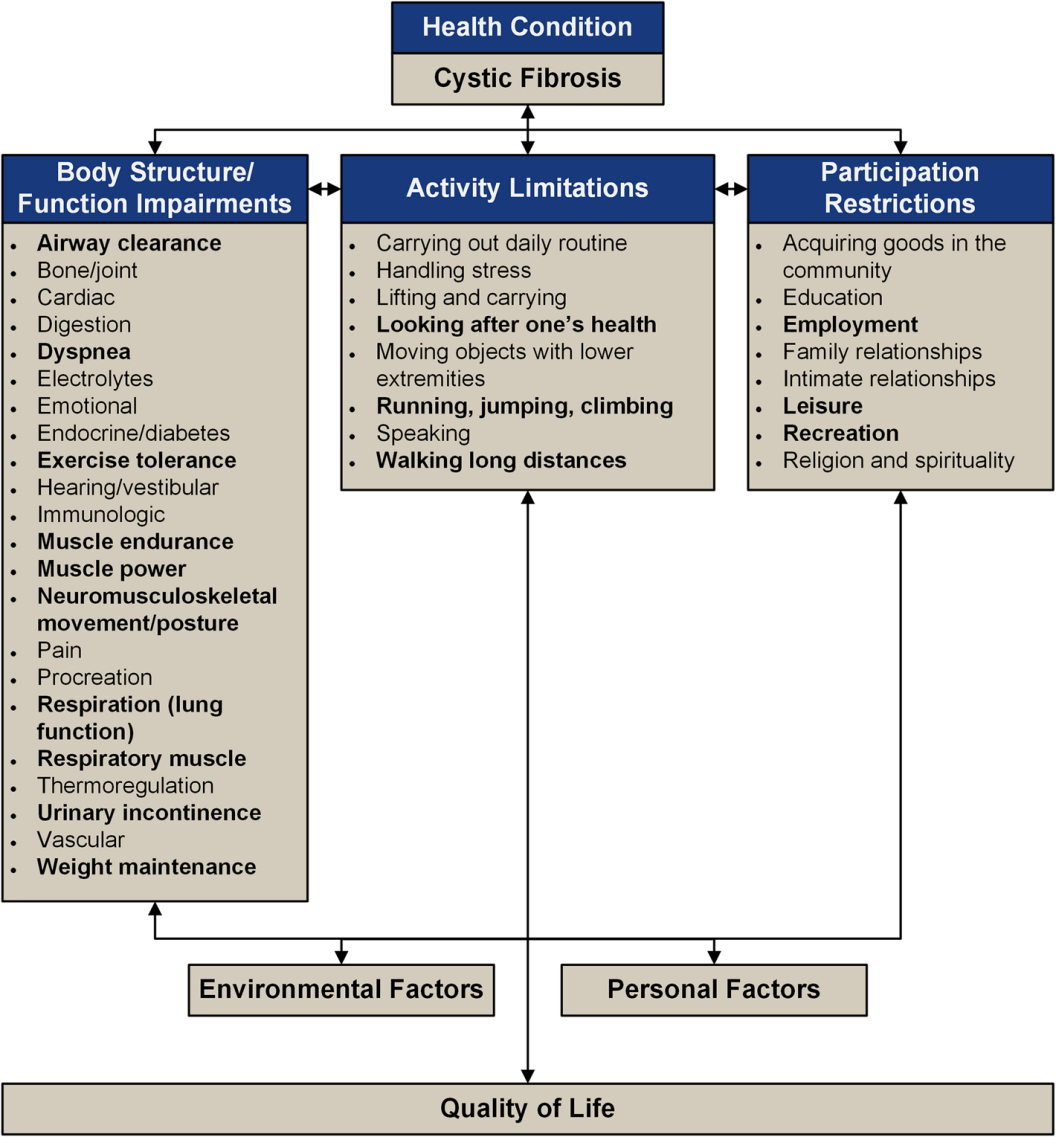
The International Classification of Functioning, Disability and Health (ICF) framework describing the interrelated body structure/function impairments, activity limitations, and participation restrictions that can occur in cystic fibrosis, limitations, and restrictions as a result of CF, and highlights (in bold) those that, in particular, are related to inspiratory muscle function. Adapted from [43]

Careful consideration of the *patient population* will be required, with future studies encouraged to control for contextual factors including, but not limited to, age, disease severity, baseline respiratory muscle performance, bacterial colonization status, modulator therapy status, aerobic fitness, body composition, rate of pulmonary function deterioration, and mutation class. Figure 1 presents the International Classification of Functioning, Disability, and Health framework on components ultimately affecting QoL in CF. Research is needed to identify which of these components are most influential in mediating and/or moderating the response to IMT, and controlling for these components should be a priority in future studies. Specific to IMT, in addition to respiratory muscle function or dysfunction (including both inspiratory and expiratory muscles), core stability and pelvic floor function may also impact the response to IMT. At present, there is a dearth of published data describing whether these factors are abnormal in CF; nevertheless, these factors may still influence respiratory muscle performance and should therefore be accounted for in future studies of IMT in CF. Additionally, IMT may also improve both core stability and pelvic floor function suggesting these may be important outcome factors to include in future studies as well. In the meantime, the use of strict inclusion and exclusion eligibility criteria will ensure a more homogeneous sample population and thereby address at least some of these components. In addition, the development of a standardized method of assessing, and subsequently grading, disease status and severity according to phenotypic determinants of respiratory muscle impairments would facilitate a more targeted, personalized approach to IMT, acknowledging that IMT may not benefit all with CF to the same degree.

Identifying the most suitable *IMT protocol* presents another significant challenge for IMT studies, with further research warranted to distinguish the most salient merits associated with the different types of resistive loading, including pressure-threshold and flow-resistive loading, as well as volume loading (i.e., voluntary hyperpnea) [27]. Importantly, the optimal training volume and intensity for IMT protocols must be elucidated; we must seek to move away from the reliance on a “one-size-fits-all” approach. Indeed, due to the phenotypic heterogeneity in those with CF, innovative IMT programs that provide a sufficient training dose to elicit specific physiological adaptations in the respiratory muscles need to be developed. For example, a patient who exhibits significant respiratory muscle weakness but preserved respiratory muscle endurance should receive a markedly different training protocol compared to a patient with preserved respiratory muscle strength but diminished respiratory muscle endurance. Furthermore, it is likely that some of those with CF, particularly those who experience a pseudo-training effect as a result of chronic airway obstruction, may require a higher training workload compared with healthier counterparts. Indeed, analogous findings have been reported in healthy elite swimmers in whom the habitual exposure to an increased WOB, due to the hydrostatic pressure the water exerts on the thorax, ameliorates the potential benefits typically engendered by IMT [44], an effect not found with lower exposures to this increased WOB [45, 46]. Alternatively, some individuals with CF may simply not benefit from IMT. For example, it is possible that particularly in the presence of mild disease and preserved respiratory muscle function, IMT may not be of any additional benefit to the current standard of care. The ergogenic effect of strength training on limb locomotor muscles appears to be optimized when athletes perform strength training to task failure [47–49]. Therefore, the development of CF-specific IMT protocols that progress to task failure (e.g., failure to generate a given inspiratory pressure during IMT) may provide the greatest potential therapeutic benefit of IMT in those with CF [50]. IMT performed at 100% of *P*_Imax_ has been shown to decrease exercising heart rate and perceived exertion, whereas IMT performed at 80% of *P*_Imax_ did not produce any such adaptations [51]. Presently, the test of incremental respiratory endurance (TIRE) regimen, which consists of a series of training breaths with a decreasing work-rest ratio, may be the best example of such an IMT protocol [22, 24, 27, 52–54]. Specifically, this type of training prescription may be better suited to inducing respiratory muscle adaptations in those with CF compared with protocols simply requiring a fixed number of breaths in each training session. Similarly, adopting a “high-intensity interval training” (HIIT)-like prescription of IMT may also enhance the potential efficacy and sustainability of this training modality, as HIIT has been shown to be effective in inducing similar training adaptations to those observed following whole-body exercise training in a more time-efficient manner.

Most IMT studies report pulmonary function-related *outcome measures* such as FEV_1_ and FVC, as well as some respiratory muscle performance parameters such as maximal inspiratory and expiratory mouth pressures (*P*_Imax_ and *P*_Emax_, respectively), to gauge respiratory muscle strength. However, these measures alone may not be sufficient to evaluate the efficacy of IMT on enhancing respiratory muscle performance [52]. Employing additional outcome measures to detect physiological adaptations, such as diaphragm and external intercostal muscle hypertrophy and motor unit recruitment pattern changes, will be informative as to whether IMT is effective in inducing true training adaptations. However, it is important to be cognizant that many such measures are not in routine clinical use and are therefore likely to be unfamiliar to people with CF and their interdisciplinary care teams. Consequently, these data may not be useful for many practitioners as they are unable to draw meaningful conclusions. Thus, these additional measures should be considered in conjunction with functional and clinical measures, such as respiratory muscle economy, ventilatory efficiency, breathing patterns, dyspnea, QoL, and participant burden.

Finally, and of utmost importance, is not whether IMT can improve respiratory muscle performance per se, but whether these improvements can transfer to more meaningful, patient-centered clinical outcomes such as those from maximal cardiopulmonary exercise testing (e.g., aerobic capacity, peak ventilation, ventilatory threshold, ventilatory efficiency) [55], health-related QoL, morbidity, health-care utilization, and even mortality. This interdisciplinary, holistic approach will allow for a more complete understanding of the physiological adaptations and subsequent clinical outcomes that can result from IMT in those with CF. This is particularly important given that those with CF already face a high burden of care [56]. Therefore, identifying whether IMT provides additive or unique benefits distinct from exercise training alone will aid in establishing whether IMT has value in the standard of care for individuals with CF.

## Conclusion

The potential for IMT to be a useful therapeutic tool in CF care remains equivocal. The present lack of quality empirical evidence supporting or refuting the use of IMT in CF highlights the challenges of designing and conducting IMT studies in CF. Future research should seek to address these challenges. Specifically, more thorough and complete reporting of important demographic information, including considerations of pertinent clinical outcome data, as well as an assessment of targeted training effects of IMT should provide a more comprehensive understanding of its efficacy in those with CF. Similarly, developing a standardized method for assessing and quantifying respiratory muscle (dys) function in order to individualize IMT training programs in CF is of utmost importance. Finally, future research should consider whether the potential benefits of IMT outweigh the additional burden placed in those living with CF.

## Data Availability

Data sharing is not applicable to this article as no datasets were generated or analyzed during the current study.

## Abbreviations

CF: Cystic fibrosis
*CFTR*: Cystic fibrosis transmembrane conductance regulator
COPD: Chronic obstructive pulmonary disease
FEV_1_: Forced expiratory volume in 1 second
IMT: Inspiratory muscle training
*P*_Emax_: Maximal expiratory mouth pressure
*P*_Imax_: Maximal inspiratory mouth pressure
QoL: Quality of life
TIRE: Test of incremental respiratory endurance
WOB: Work of breathing
